# Differential regulation of abundance and deadenylation of maternal transcripts during bovine oocyte maturation in vitro and in vivo

**DOI:** 10.1186/1471-213X-7-125

**Published:** 2007-11-07

**Authors:** Aurore Thélie, Pascal Papillier, Sophie Pennetier, Christine Perreau, Juan Martin Traverso, Svetlana Uzbekova, Pascal Mermillod, Catherine Joly, Patrice Humblot, Rozenn Dalbiès-Tran

**Affiliations:** 1 INRA, UMR85 Physiologie de la Reproduction et des Comportements, F-37380 Nouzilly, France ; CNRS, UMR6175,Nouzilly, France, F-37380 ; Université de Tours, F-37041 Tours, France ; Haras Nationaux, Nouzilly, France, F-37380; 2 Centre de Recherche en Biologie de la Reproduction, Département des Sciences Animales, Université Laval, Québec, Canada, G1K 7P4; 3 Union Nationale des Coopératives d'Elevage et d'Insémination Animale, station UNCEIA/UCEAR, Chateauvillain, France, F-38300; 4 Union Nationale des Coopératives d'Elevage et d'Insémination Animale, Département R&D, Maisons-Alfort, France, F-94703

## Abstract

**Background:**

In bovine maturing oocytes and cleavage stage embryos, gene expression is mostly controlled at the post-transcriptional level, through degradation and deadenylation/polyadenylation. We have investigated how post transcriptional control of maternal transcripts was affected during in vitro and in vivo maturation, as a model of differential developmental competence.

**Results:**

Using real time PCR, we have analyzed variation of maternal transcripts, in terms of abundance and polyadenylation, during in vitro or in vivo oocyte maturation and in vitro embryo development. Four genes are characterized here for the first time in bovine: ring finger protein 18 (*RNF18*) and breast cancer anti-estrogen resistance 4 (*BCAR4*), whose oocyte preferential expression was not previously reported in any species, as well as Maternal embryonic leucine zipper kinase (*MELK*) and *STELLA*. We included three known oocyte marker genes (Maternal antigen that embryos require (*MATER*), Zygote arrest 1 (*ZAR1*), NACHT, leucine rich repeat and PYD containing 9 (*NALP9*)). In addition, we selected transcripts previously identified as differentially regulated during maturation, peroxiredoxin 1 and 2 (*PRDX1, PRDX2*), inhibitor of DNA binding 2 and 3 (*ID2*, *ID3*), cyclin B1 (*CCNB1*), cell division cycle 2 (*CDC2*), as well as Aurora A (*AURKA*). Most transcripts underwent a moderate degradation during maturation. But they displayed sharply contrasted deadenylation patterns that account for variations observed previously by DNA array and correlated with the presence of a putative cytoplasmic polyadenylation element in their 3' untranslated region. Similar variations in abundance and polyadenylation status were observed during in vitro maturation or in vivo maturation, except for *PRDX1*, that appears as a marker of in vivo maturation. Throughout in vitro development, oocyte restricted transcripts were progressively degraded until the morula stage, except for *MELK *; and the corresponding genes remained silent after major embryonic genome activation.

**Conclusion:**

Altogether, our data emphasize the extent of post-transcriptional regulation during oocyte maturation. They do not evidence a general alteration of this phenomenon after in vitro maturation as compared to in vivo maturation, but indicate that some individual messenger RNA can be affected.

## Background

The fully grown oocyte (FGO) ability to sustain genome reprogramming and embryo development is associated with a specific and complex pattern of gene expression. This has prompted high-throughput studies of the mammalian oocyte transcriptome in mouse [1,2], in human [3] or bovine [4]. In most tissues, the transcriptome reflects the contemporary pattern of RNA synthesis and protein synthesis. In this respect, the fully grown and maturing oocytes are atypical models, since at these stages the oocyte is essentially transcriptionally quiescent. Rather, the FGO transcriptome results from active RNA synthesis during the oocyte growth phase, followed by post-transcriptional regulation. A subset of maternal transcripts undergoes post-transcriptional regulation through shortening of the polyA tail, and is stored protected from degradation and from the translation machinery. At the time when the protein is required, during maturation, fertilization or the early stages of embryo development, such dormant transcripts can undergo cytoplasmic polyadenylation and support translation, then are rapidly degraded. The underlying molecular mechanisms were shown to involve *cis *regulatory elements within the 3' untranslated region, including cytoplasmic polyadenylation elements (CPE) (for reviews, see [5-7]). However, during maturation most maternal transcripts are expected to follow a default deadenylation and degradation pathways [8-10].

Besides a complex post-transcriptional regulation pathway, the mammalian oocyte transcriptome is characterized by specific transcripts not found in somatic tissues, as first evidenced in mouse [11-13]. The hypothesis that so-called oocyte-specific genes would be important for fertility was confirmed by functional genomics experiments in mouse (reviewed in [12]). A first class of genes is required for normal folliculogenesis and fertilization. Other genes are maternal effect genes, i.e. they are dispensable until fertilization but they are required for proper embryo development: most embryos from mothers null for Zygote arrest 1 (*Zar1*), Maternal antigen that embryos require (*Mater*, official symbol *Nalp5 *as a member of the NACHT, leucine rich repeat and PYD containing, (NALP) family), or *Stella *(official name Developmental pluripotency associated 3 (*Dppa3*)) are blocked before the blastocyst stage [14-17]. Functional importance of other oocyte-enriched genes remains to be assessed, such as Oocyte secreted protein 1 (*Oosp1*), Maternal embryonic leucine zipper kinase (*Melk*), the Oogenesin genes and several *Nalp *family members [13,18-21] (Studies in bovine have identified orthologous genes, as well as potentially novel oocyte-specific genes in this species [22-27].

The main objective of this study was to investigate post-transcriptional regulation of maternal transcripts during bovine oocyte maturation in relation with its quality, i.e. its ability to undergo meiosis and sustain embryo development. Bovine is definitely a pertinent scientific model to analyze the relationship between oocyte developmental competence and the regulation of maternal transcripts. First, embryonic genome major activation is delayed up to the 8/16 cell stage about 3.5 days after fertilization, whereas in mouse it is already completed 24 hrs after fertilization at the end of the 2-cell stage. This chronology highlights the importance of timely control of maternal RNA. Most importantly, several physiological and experimental models of oocyte quality have been defined in this species (reviewed in [28]). In particular, in vivo matured oocytes present the highest developmental competence (as estimated by the blastocyst rate), while in vitro maturation remains a suboptimal alternative [29-32]. As nuclear maturation is not affected, the difference is usually attributed to an inadequate reproduction of the cytoplasmic features of oocyte maturation in vitro.

Here, using real time PCR, we have analyzed the variation of maternal transcripts during bovine oocyte maturation and embryo development, in terms of abundance and polyadenylation. We selected genes likely involved in oocyte quality, based on their restricted expression in the germ cell or their previous isolation as markers of maturation [33]. Three oocyte specific genes (*MATER*, *NALP9 *and *ZAR1*) were previously identified. Four additional genes are characterized here for the first time: the bovine *MELK *and *STELLA *genes, as well as two genes whose oocyte preferential expression was not previously reported in any species, ring finger protein 18 (*RNF18*) and breast cancer anti-estrogen resistance 4 (*BCAR4*). We also examined the cell-cycle regulator Aurora A (*AURKA*) whose transcript is abundant in meiotically arrested oocytes. In addition, we followed 6 transcripts previously identified as down- or up-regulated during oocyte maturation: cyclinB1 (*CCNB1*) and cell division cycle 2 (*CDC2*) encoding components of the M-phase promoting factor, as well as peroxiredoxin (*PRDX*) 1 and 2, and inhibitor of DNA binding (*ID*) 2 and 3 [33]. Overall, our results show a moderate degradation of most transcripts during maturation, and evidence contrasted deadenylation patterns that mostly account for variations observed previously by DNA array. As a differential model of developmental competence, we compared regulation of maternal transcripts during in vitro and in vivo maturation. During embryonic development, distinct profiles were observed, and most oocyte marker genes remained silent after embryonic genome activation.

## Results

### Characterization of four bovine genes preferentially expressed in the oocyte

We characterized transcripts represented by five distinct EST in our library of EST preferentially represented in bovine oocyte as compared to somatic tissues [23]. One EST presented a high sequence homology with the human *MELK *gene, while four others did not return a match. Virtual Northern blots were run and hybridized using the considered EST as probes. Then, full length transcript sequence was determined by Rapid Amplification of cDNA Ends (RACE). Gene structure and chromosomal localization was deduced by aligning the full length cDNA and the bovine genome sequences. We were able to identify the three remaining genes as *STELLA*, *RNF18 *and *BCAR4*.

#### MELK

**Figure 1:**
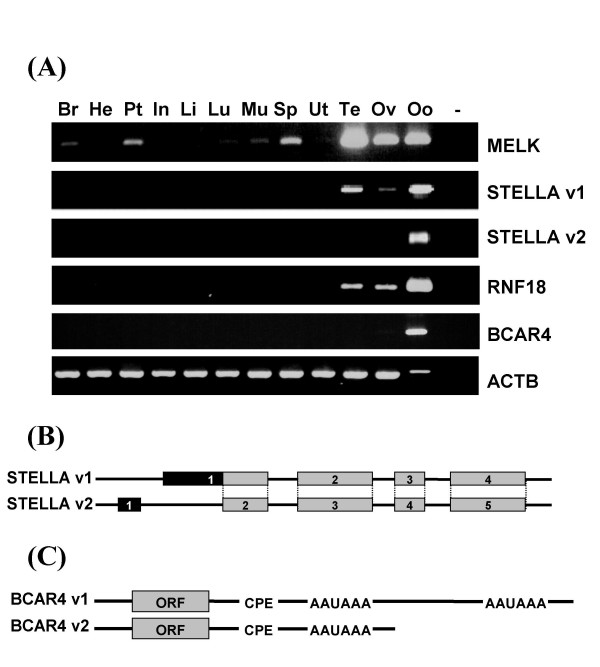
**Characterization of oocyte specific transcripts**. (A) Expression pattern of *MELK*, *RNF18*, *BCAR4 *and *STELLA *in bovine somatic and gonadic tissues by RT-PCR. Tissues are indicated above the lanes: brain (Br), heart (He), pituitary gland (Pt), intestine (In), liver (Li), lung (Lu), muscle (Mu), spleen (Sp), uterus (Ut), testis (Te), Ovary (Ov), oocyte (Oo); no PCR substrate as negative control (-). RT-PCR for ACTB is shown as a positive control. An estimated 500-fold higher amount of substrate was used in PCR for somatic tissues and gonads as compared to the oocyte. (B) Schematic representation of STELLA transcript variants generated by alternative splicing. (C) Schematic representation of BCAR4 variants generated by alternative use of two polyadenylation signals (AAUAAA).

One EST was 88% homologous to human *MELK*. As observed by RT-PCR, *MELK *is not really oocyte specific; rather, it is expressed at low level in most tissues, but strongly expressed in both the male and female gonads and in the oocyte (Fig 1A). A virtual Northern blot revealed a single 2.5 kbp cDNA (not shown). The full length 2445 bp sequence deduced from RACE (genbank accession hoEF446902) is assembled from 18 exons, including a first non coding exon that is lacking in the bovine *MELK *transcript that has since been predicted within the genome onto BTA8. The full-length sequence contains a 1953 nucleotide long open reading frame (ORF), encoding a predicted 650 aa/74 kD protein wse sequence is 90% and 80% identical to its human and mouse counterparts, respectively; it presents similar serine/threonine kinase domain and kinase associated domain at the N-terminus and C-terminus respectively.

#### STELLA

Two independent EST initially could not be identified based on their sequence, including previously described EST5B4 [23]. By virtual Northern blot we observed a doublet: the corresponding 1590 and 1347 bp long cDNA, as deduced from RACE, are generated by alternative splicing at their 5' end (Fig 1B) (genbank accession EF446904, EF446905). They present the same 492 nt ORF encoding a 163 aa putative protein. We identified a bovine orthologue of the germ cell marker Stella, despite limited sequence homology with the human and mouse counterparts (30% and 26% respectively). By RT-PCR, both variants were undetected in somatic tissues. Variant 1 was detected in testis, ovary and oocyte, while variant 2 was detected only in oocytes (Fig 1A).

#### RNF18

Initially our EST could not be identified by sequence homology with sequences in public databases. By RT-PCR, the transcript was specifically detected in testis, ovary and oocyte (Fig. 1A). A 1723 bp cDNA, observed by virtual Northern blot, was cloned by RACE (genbank accession EF446903). The 1353 nt long ORF encodes a 450 aa/52 kD putative protein with 3 major regions: a N-terminal ring finger, a B-box, and a C-terminal SPRY domain also found in ryanodine receptor. This structure identifies the protein as a member of the tripartite motif (TRIM) family. The corresponding locus LOC525113 on BTA29 has since been predicted from the bovine genome with the annotation "similar to ring finger protein 18 (Testis-specific ring-finger protein)", whose human orthologue was found primarily expressed in testis [34] and is also named *TRIM49*. Identity is confirmed by analogous gene structure, with six coding exons of similar length in human and bovine. As compared to the predicted bovine transcript, our cDNA contains three additional exons, including a 5' non coding exon and two short coding exons.

#### BCAR4

Two transcripts (genbank accession EF446906, EF446907) observed by virtual Northern blot were cloned by RACE starting from our unidentifiable EST. They are 638 and 1317 bp long respectively, and are generated through the alternative use of two polyadenylation signals, downstream a putative cytoplasmic polyadenylation element (Fig. 1C). The 363 nt long ORF encodes a putative 120 aa/14 kD protein. The bovine gene is located onto BTA25, between two loci annotated "similar to zinc finger CCCH-type domain containing 7" and "ribosomal L1 domain containing 1"respectively. By conserved synteny we identified the human ortholog breast cancer anti-estrogen resistance 4 (*BCAR4*). Both the bovine and human ORF are included within a single exon, and encode proteins with two putative transmembrane domains; yet they do not display significant sequence identity. In human, ESTs have been isolated mainly from placenta, and to our knowledge expression of *BCAR4 *has not been reported in the gonads or the oocyte. By RT-PCR the transcript was detected specifically in the ovary at trace level and in the oocyte (Fig. 1A).

### Variation of maternal transcripts during in vitro maturation

**Figure 2:**
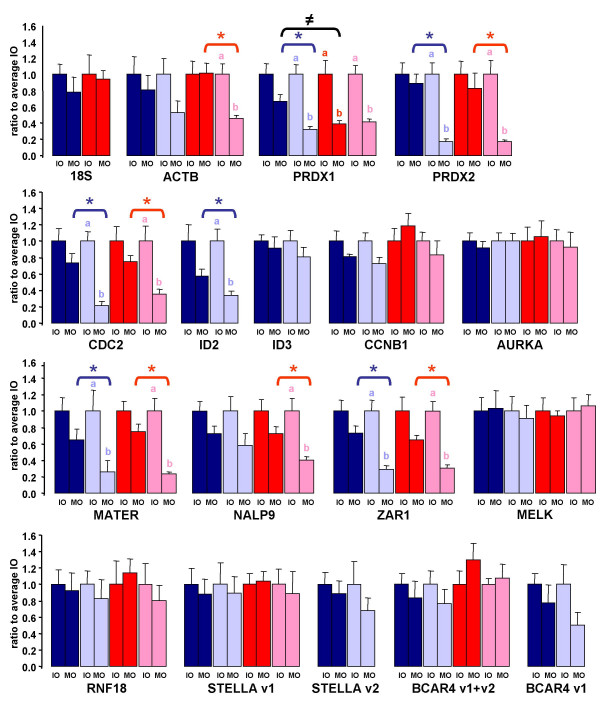
**Variation of maternal transcripts during in vitro and in vivo maturation**. Variation of transcripts total forms (dark color bars) or polyadenylated forms (light color bars) during in vitro (blue) or in vivo (red) oocyte maturation. The ratio to the mean value in immature oocytes is represented (mean ± SEM). IO and MO refer to immature oocytes and mature oocytes respectively. For each gene and form, different letters indicate a significant difference between IO and MO; an asterisk indicates a significant difference between variation of total and polyadenylated forms; "≠" indicates a significant difference between in vitro and in vivo maturation (P < 0.05).

We followed the variation of selected maternal transcripts during in vitro oocyte maturation, in terms of abundance and polyadenylation. Real-time PCR was run onto random hexamer-primed RT products, in order to estimate their abundance, and oligo(dT)_15_-primed RT products, which are affected by both abundance and polyadenylation. For each transcript, the ratio to the mean value in immature oocytes is represented (Fig 2). For most transcripts, including 18S ribosomal RNA, the abundance did not vary significant during maturation, although an average 20% decrease was observed. *PRDX1*, *ID2 *and *MATER *transcripts appeared the most affected (over 33% degraded). As expected due to transcription repression during maturation, no transcript displayed a significant increase. The patterns of polyadenylated transcripts revealed sharp contrasts. *CDC2*, *PRDX1*, *PRDX2*, *ID2*, *MATER, ZAR1 *transcripts were strongly affected, as detection level dropped below one third of their initial level. For all these genes, variations of the total and polyadenylated forms were significantly different during maturation (Fig. 2, *). For *NALP9 *and *BCAR4 *variant 1, the decrease (41 and 49% respectively) was still more important than the decline observed for the total form (27 and 23%), but the difference was not statistically significant. On the other hand, seven genes appeared hardly affected; the polyadenylated transcript followed a parallel evolution as the total form, and decreased by less than 30%. These were *AURKA*, *CCNB1*, *ID3*, *MELK*, *RNF18*, *STELLA *(two variants analyzed individually), and *BCAR4 *(both variants analyzed together).

### Comparison of in vitro maturation and in vivo maturation

In order to compare the variation of maternal transcripts during in vitro and in vivo maturation, a subset of genes was followed in oocytes collected by ovum pick up (OPU) (Fig. 2). *BCAR4 *variant 1, *STELLA *variant 2, *ID2 *and *ID3 *transcripts were not quantified in these minute samples due to requiring more substrate. Again, for most transcripts the total form displayed a limited and statistically non significant variation during maturation. An exception was *PRDX1*, whose detection level dropped by 62% during in vivo maturation: this was statistically different from both the level in immature oocytes and the 33% drop during in vitro maturation (Fig. 2, ≠). As observed during in vitro maturation, polyadenylated transcripts displayed more contrasted patterns. ACTB, PRDX1, PRDX2, CDC2, MATER, NALP9, ZAR1 decreased significantly (Fig. 2, *). Indeed, for polyadenylated transcripts, the mean values were often remarkably close after in vitro and in vivo maturation. Interestingly, *PRDX1 *polyadenylated form displayed a similar variation in both cases.

### Profile of maternal transcripts during preimplantation development

**Figure 3:**
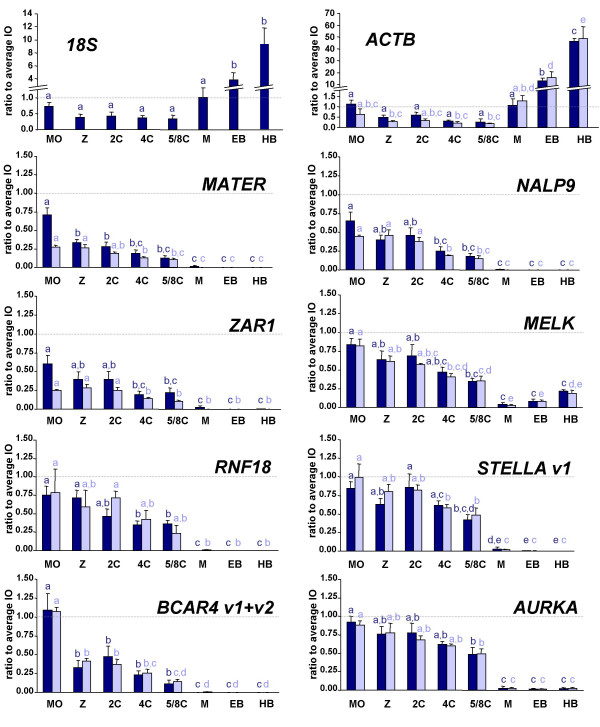
**Profile of transcripts throughout embryo development**. Analysis of transcripts total forms (dark blue) and polyadenylated forms (light blue) during early embryo development: in vitro matured oocyte (MO), in vitro cultured zygote (Z), 2-, 4-, 5/8-cell embryos, morula (M), expanded blastocysts (EB) and hatched blastocysts (HB). The ratio to immature oocytes (set at 1, dotted line) is shown (mean ± SEM). Within the profiles of the total form or polyadenylated form, different superscripts indicate a significant difference (P < 0.05).

Then we analyzed the variation of oocyte markers during the early stages of embryo development (Fig. 3). As examples of housekeeping genes, we quantified *18S *and *ACTB *transcripts. Their levels started to increase between the 5/8-cell and morula stages, as expected since major genome activation occurs in bovine at the 8/16-cell stage, and rose further during blastocyst formation and hatching. By contrast, detection of all oocyte markers decreased between the 5/8-cell and morula stages, and all but *MELK *decreased further in blastocysts, often below detection level. *MELK *was indeed activated in blastocysts.

By the zygote stage, most transcripts (*18S*, *ACTB*, *MATER*, *NALP9*, *ZAR1*, *BCAR4*) had fallen below half their level in immature oocytes. Four transcripts remained fairly abundant in 2-cell embryos, i.e. over 60% of their initial level: *MELK*, *RNF18*, *STELLA *variant 1 and *AURKA*, whose level decreased progressively throughout pre-MET development.

Finally, we did not observe significant differences between the total and polyadenylated forms of any transcript during embryo development from zygote to hatched blastocysts.

### Regulatory elements in the 3' untranslated region

We searched the 3' untranslated region of the transcripts for some of the regulatory sequences that are known to be involved in the post-transcriptional regulation of maternal RNA in model species. There is no unique consensus sequence for the CPE, but rather variants from the basic U_4–5_A_1–2_U sequence were identified in xenopus and mouse within 100 nucleotides upstream the nuclear polyadenylation signal [5,9,35] Based on this definition, potential CPE-like sequences were found in *ID2*, *ID3*, *CCNB1*, *MELK*, *RNF18*, *STELLA *and *AURKA *(Table 1). Except for ID2, their total and polyadenylated forms were not differentially regulated during in vitro or in vivo maturation (Fig. 2, no *). Reciprocally, no such CPE could be recognized within the 3' untranslated region of *CDC2*, *PRDX1*, *PRDX2*, *MATER*, *NALP9 *and *ZAR1 *transcripts, or much farther from the nuclear polyadenylation signal for *ACTB*; their polyadenylated forms all displayed a significant decline as compared to their total form during in vivo maturation (Fig. 2, *), as well as in vitro maturation although below significance threshold for *NALP9 *and *ACTB*. CPE-like sequences were present in *BCAR4 *transcripts, either close to the nuclear polyadenylation signal for variant 2, or 600 nucleotides upstream for variant 1 (Table 1). BCAR4 variant 1 exibited a similar profile as NALP9 during in vitro maturation. When both variants were analysed together, neither the total nor the polyadenylated transcripts appeared much affected (Fig. 2). Of note, *STELLA *and *ACTB *contained U_10_CU_8 _and U_12_, U_15 _and U_11 _sequences respectively. These might be embryonic CPE, i.e. elements that target transcripts for cytoplasmic polyadenylation after fertilization [6]. Yet no such polyadenylation was evidenced by real-time PCR.

**Table 1:** putative regulatory elements and their position relative to the nuclear polyadenylation signal (NPS)

**gene (transcript variant)**	**CPE-like sequence (distance from NPS)**	**Class I/II ARE-like motif (distance from NPS)**
*ACTB*	UUUUUAAU (340 nt)	AUUUA (447 nt)
*AURKA*	UUUUAAAU (5 nt)	none
*BCAR4 *(v1)	UUUUUAU (633 nt)	none
*BCAR4 *(v1)	UUUUAAU (686 nt)	
*BCAR4 *(v2)	UUUUAAU (12 nt)	none
*CCNB1*	UUUUAAU (overlapping)	AUUUAUUUA (5 nt)
*CDC2*	none	AUUUA (57 nt)
*ID2*	UUUUUAU (35 nt)	AUUUA (402 nt, 295 nt, 163 nt)
*ID3*	UUUUUAU (19 nt)	AUUUA (85 nt)
*MATER*	none	none
*MELK*	UUUUUAAU (17 nt)	AUUUA (87 nt)
*MELK*	UUUUUAAAU (106 nt)	
*NALP9*	none	none
*PRDX1*	none	none
*PRDX2*	none	none
*RNF18*	UUUUAU (3 nt)	AUUUA (overlapping with CPE)
*STELLA*	UUCUAAU (24 nt)	AUUUA (536 nt)
*ZAR1*	none	none

We also searched the 3' untranslated region for so called A/U rich element (ARE) that can target corresponding transcripts for active deadenylation, as opposed to the default deadenylation pathway (Table 1). Although there is no strict consensus sequence, AREs have tentatively been grouped into three major classes [36,37] Class I and II ARE contain one or several dispersed or adjacent copies of AUUUA motifs in a U-rich context; such motifs were found in bovine *ACTB*, *CDC2*, *ID2*, *ID3*, *MELK*, *RNF18*, *STELLA *and *CCNB1 *(Table 1). Class III ARE are described as over 50 nucleotide long U-rich regions; several transcripts may fall into this category. Finally, the presence of multiple UGU motif in a U/G-rich context has also been reported to target mRNA for deadenylation [38]; several bovine transcripts indeed contained at least 4 copies of the triplet within 45 nucleotides. But altogether, no obvious correlation appeared between the presence of putative ARE motifs or UGU-rich sequences and the transcripts profile during maturation or development.

## Discussion

In this study, we have characterized four novel bovine genes with a preferential oocyte expression. Then, using real-time PCR, we have analysed these and other maternal transcripts for changes in their abundance and polyadenylation during in vitro or in vivo maturation as well as in vitro development up until the hatched blastocyst stage.

### Influence of polyA tail onto transcripts detection by real time PCR and microarrays

A few studies have commented on the different apparent transcript levels deduced from real time PCR onto cDNA synthesized from oligo(dT) or random primers in bovine oocytes and embryos [39-41] Here we extend this observation to a number of oocyte-specific or non specific transcripts. The discrepancy is attributed to variations in the polyA tail, which is of major interest in this model due to the predominantly post-transcriptional regulation of gene expression in FGO and pre-MET embryos. We may also suspect a similar bias when polyadenylated RNA are purified [42-45] Indeed, the influence of polyA tail has consequences beyond the analysis of real time PCR data, into the field of embryogenomics. Due to limited biological samples, amplified probes are usually generated from mammalian oocytes/embryos for hybridizing onto microarray, through protocols that involve reverse transcription from oligo(dT). Therefore, a similar bias associated with the polyA tail is expected in these transcriptomic analyses. Although this had been suggested before, experimental validation analyzing both the total and polyadenylated transcripts was lacking. In this study we have included six genes previously identified by DNA array as markers of oocyte maturation: *CDC2*, *PRDX1 *and *PRDX2 *transcripts were shown to be negatively regulated, while *CCNB1*, *ID2 *and *ID3 *were positively regulated [33]. Here, using real time PCR, we observed only a minor degradation of five transcripts (all but *ID2*) during maturation. But we did observe a dramatic decrease in polyadenylated transcripts level for *CDC2*, *PRDX1 *and *PRDX2*, while *ID3 *and *CCNB1 *polyadenylated transcripts appeared hardly affected. These results indeed confirm that the array experiment was influenced by the polyadenylation status of these transcripts. Similarly, down regulation of MATER during oocyte maturation, as observed on a microarray [46], must reflect the significant decrease of the polyadenylated transcript evidenced here. In our experiment, *ID2 *did not follow the expected pattern: while among up-regulated transcripts revealed by DNA array screening, the polyadenylated transcript displayed a significant decline when assessed by real time PCR. We have currently no satisfying explanation for this discrepancy. Hybridization of a non-specific probe due to cross-species hybridization appears unlikely due to the high sequence conservation (94%) between the bovine and human coding sequences. Alternatively, a different transcript variant might have hybridized onto the array than the one studied by real time PCR.

Altogether, our results should prompt to reconsider interpretation of DNA array and real-time PCR data in models with potential regulation of the polyA tail. In mouse and bovine oocytes and embryos, array validation experiments were based on reverse-transcription initiated either from oligo(dT) [44,45,47] or from a mix of both oligo(dT) and random hexamers [48]. In order to minimize the impact of variable polyA tails, a modified protocol for probe amplification was recently developed [49]. On the other hand, detecting transcripts polyadenylation status on an array may be viewed as an asset, since it provides a footprint of mRNA that can be translated at a given stage, circumventing the need for purifying polyadenylated transcripts [50].

### Selective deadenylation of maternal transcripts during in vitro maturation

Maturation-associated degradation of stored maternal transcripts has been known for a long time. In the mouse, earlier studies reported a 19% decrease in RNA content, as measured by ethidium bromide fluorescence; most of it was accounted for by the degradation of rRNA [51,52] Here, bovine *18S *ribosomal RNA was found to decrease during in vitro maturation by 22% (not statistically significant), a moderate degradation that could not be observed previously by Northern blot [39]. Our value appears fairly close to the value reported in the mouse. Yet, based on different developmental kinetics in both species, one might have expected a lesser extent of degradation in the bovine, as maternal rRNA will be required to sustain translation in embryo for several days versus only a few hours in mouse. A higher absolute RNA content in bovine oocytes as compared to mouse oocytes [53] might compensate for this similar global degradation rate.

Non ribosomal RNA is also affected, but the destruction of transcripts during maturation in mice appears a selective rather than promiscuous process: this was observed on individual genes in earlier studies [54] and was recently confirmed using a microarray [49]. Here, most transcripts displayed a limited degradation during maturation. Within our set of genes, *MATER*, *PRDX1 *and *ID2 *transcripts were most affected, over 33% degraded. Degradation of mouse *Mater *transcript in ovulated oocytes was also recently reported [49]. Contrasted profiles were observed for the polyadenylated forms. In this respect, oocyte specific transcripts did not exhibit a uniform or a particular pattern; a similar heterogeneity was observed for housekeeping genes [40]. For a few genes, protein data are available. During in vitro maturation, the level of CDC2 and MATER proteins was not much altered as observed by Western blot [55,56], while an isoform of PRDX2 was shown to be synthesized through a proteomics approach [57]. Here, all three polyadenylated transcripts displayed a major decline. This apparent discrepancy may only reflect complex post-transcriptional and translational control, and illustrates the uncoupling of transcriptome and proteome in FGO and mature oocytes.

### Comparison of in vitro and in vivo maturation

Whether considering variation of total or polyadenylated transcripts, for most genes we did not evidence a different pattern after in vitro vs in vivo maturation. Indeed, for polyadenylated transcripts mean values were often remarkably close. Overall, our data support the notion that degradation and deadenylation are not severely affected by in vitro maturation as compared to in vivo maturation. Lower developmental competence of in vitro matured oocytes should not be attributed to a general alteration of post-transcriptional regulation of maternal transcripts. Yet the following points have to be kept in mind. First, we considered only relative variations between immature and mature oocytes; should a transcript be more abundant at both stages in one model (in vitro or in vivo maturation), the corresponding ratio might not be affected. Second, we collected oocytes before and after maturation, but not in the course of maturation, when transient differences might have been revealed. Third, it can not be excluded that superovulation treatment affects oocyte transcriptome, so that OPU oocytes may not reflect normal physiology.

An intriguing exception was *PRDX1*, whose total form declined more severely during in vivo as compared to in vitro maturation. This transcript was reported to increase in oocytes from growing follicles over 8 mm in diameter [58]; however, direct comparison with our own results is difficult since in this paper, there is no mention of whether transcript total form or only polyadenylated form was quantified, and data were normalized to an internal rather than an exogenous reference. In our study, immature oocytes were meant to be collected both in vivo and post-mortem from follicles under 8 mm, and thus the follicle size should not impact our data. Altogether, these studies may indicate that *PRDX1 *level in oocytes is particularly sensitive to environmental changes during late folliculogenesis and maturation. Analysis in other physiological models will reveal whether it is a general marker of bovine oocyte developmental competence.

In a previous study, a correlation between shorter polyA tail and low developmental competence had been reported; the difference in polyA tail could be observed both before and after in vitro maturation [59]. The authors considered a different model of oocyte quality, based on the morphology of the ovary: their competent oocytes, fairly similar to our post mortem collected oocytes, were compared with oocytes with really poor developmental (5% blastocyst rate) [60].

### Profile of maternal transcripts during embryo development

Profiles during embryo development reflected the degradation of maternal transcripts between the immature oocyte and the 5/8-cell stage. In morulae and blastocysts, detection of *18S *and *ACTB *increased, reflecting embryonic genome activation. Strikingly, oocyte markers continued to decline in morulae, often below detection level. Thus they were not activated at the time of major genome activation, as previously reported for *MATER*, *OOSP1*, *BMP15*, *GDF9*, *ZAR1 *and *NALP9 *[22,24,27,56] and for the vast majority of mouse oocyte specific genes [2]. All but *MELK *remained silent in expanded and hatched blastocysts. Interestingly, *Melk *is also activated in mouse 8-cell embryos [2,19]

Most transcripts displayed a rapid decline during the earlier stages of embryo production, i.e. oocyte maturation, fertilization and zygote formation. *BCAR4 *was fairly constant during maturation, and then dropped concomitantly with zygote formation. Whether this decline follows translation of a protein required for early embryogenesis remains to be investigated. By contrast, *MELK*, *RNF18*, *STELLA *variant 1 and *AURKA *exhibited a progressive decrease from immature oocytes to 5/8-cell embryos. Again, it is tempting to speculate that these transcripts support translation at all stages to ensure continuous production of a short half-lived protein necessary for development; but they may just as likely be degraded without supporting translation.

For all transcripts, the total and polyadenylated forms displayed parallel profiles throughout embryo development from zygote to hatched blastocyst. We did not evidence polyadenylation of a transcript at a specific stage when it would be recruited for translation. However, this may be too transient a phenomenon to be observed in a population of several embryos. Rather, individual analysis of numerous embryos might be required to reveal the few embryos containing a transcript with an elongated polyA tail.

### CPE and the control of maternal transcripts during maturation

Globally, we observed a correlation between the presence of a CPE-like sequence and the regulation of polyadenylated transcripts during maturation. The profiles of transcripts without a CPE were coherent with them following the expected default deadenylation pathway. Reciprocally, the polyadenylated forms of transcripts with a putative CPE appeared stable, protected from further deadenylation, with the exception of ID2. The divergent profile of ID2 may indicate that a CPE is necessary, but not sufficient for stabilizing the polyA tail. Alternatively, it may have been generated by a distinct transcript variant with a longer 3' untranslated region (and thus a far upstream CPE, see BCAR4).

Altogether, our results are in agreement with conclusion from a recent microarray study in *Xenopus tropicalis*: CPE were under-represented in transcripts that were deadenylated during maturation, and over-represented in transcripts that were polyadenylated during maturation or substrate for post-fertilization deadenylation [61]. Here we did not observe a significant increase that would result from cytoplasmic polyadenylation. This is not unexpected as the polyadenylated form may be transient and no longer present in mature oocytes; a time-course analysis might be necessary, especially during the first 6 to 10 hours when cytoplasmic polyadenylation is believed to initiate [62,63] Alternatively, the elongation of the polyA tail may not be sufficient to be detected by this methodology; in fact, in a previous study polyadenylation of one *CCNB1 *variant was observed using a PCR based polyA test, but not by real-time PCR [64].

## Conclusion

In this study, we have analyzed the regulation of selected maternal transcripts during bovine oocyte maturation, and its correlation with the presence of a putative CPE. As a model of differential developmental competence, we have compared maturation in vitro and maturation in vivo. The control of degradation and deadenylation did not appear globally compromised by in vitro manipulation, as only *PRDX1 *exhibited strikingly different patterns in the two conditions.

## Methods

### Post mortem oocyte collection and embryo production

Ovaries from adult cows were collected at a slaughterhouse and the oocyte-cumulus complexes were aspirated from 3–8 mm follicles, selected based on morphological criteria and washed in saline solution (for details see [65]). Denuded germinal vesicle stage immature oocytes and *in vitro *matured metaphase II oocytes (polar body extrusion was verified) were obtained as previously described [33]. Briefly, oocyte-cumulus complexes were denuded by mechanical treatment either before or after *in vitro *maturation in TCM199 (Sigma, Saint Quentin Fallavier, France) supplemented with 10 ng/ml epidermal growth factor and 10% fetal calf serum for 22 h at 39°C in water-saturated air with 5% carbon dioxide. Embryos were produced as previously described [56]. Groups of 10 embryos were collected over preimplantation development: zygotes and 2-cell embryos (day 2), 4-cell, 5 to 8-cell embryos (day 3), morulae (day 5), expanded blastocysts (day 7) and hatched blastocysts (day 8). All oocyte and embryo samples were stored frozen in RNA later (Ambion, UK).

### In vivo oocyte collection

All procedures were approved by the Agricultural and Scientific Research Government Committees in accordance with the guidelines for Care and Use of Agricultural Animals in Agricultural Research and Teaching (approval PB380). Oocytes were collected in vivo from eight superovulated Montbeliard cows by OPU at 12 or 60 h after prostaglandin injection to collect immature and in vivo-matured oocytes, respectively. Oocytes were denuded as described above, and stored frozen in RNAlater.

### Characterization of full-length cDNA

Gonads were collected from animals slaughtered in a commercial abattoir. Other organs were collected from an animal bred and killed in an experimental setting; all procedures were approved by the Agricultural and Scientific Research Government Committees in accordance with the guidelines for Care and Use of Agricultural Animals in Agricultural Research and Teaching (approval A37801). RNA was isolated from biopsies using Trizol (Invitrogen, Cergy Pontoise, France) and DNAse-treated. RACE-PCR and virtual Northern blot (i.e. Southern blot of full length cDNA) were carried out as previously described [22]. Sequences are available from Genbank and from the Ovogenae programme database [66]. Primers sequences are given in Table 2.

**Table 2:** primers sequences

**gene (transcript variant)**	**primer**	**sequence**	**Genbank accession**
*luciferase*	fwd	TCATTCTTCGCCAAAAGCACTCTG	
	rev	AGCCCATATCCTTGTCGTATCCC	
*18S*	fwd	CGGACCAGAGCGAAAGCATTTG	DQ222453
	rev	GAATAACGCCGCCGCATCG	
*ACTB*	fwd1	CGTGACATTAAGGAGAAGCTGTGC	NM_173979
	fwd2	GCGTGACATCAAGGAGAAGC	
	rev1	CTCAGGAGGAGCAATGATCTTGAT	
	rev2	TGGAAGGTGGACAGGGAGGC	
*MATER*	fwd	GCTGGAGGCGTGTGGACTG	NM_001007814
	rev	GGTCTGTAGATTAGAGGTGGGATGC	
*NALP9*	fwd	GCGGCGGTGCTGTGTGAAG	NM_001024664
	rev	CTGCGTCTGCCCTCGTCATC	
*ZAR1*	fwd	TGCCGAACATGCCAGAAG	NM_001076203
	rev	TCACAGGATAGGCGTTTGC	
*MELK*	fwd1	CCTTGATGAAGATTGTGTAACGGAAC	EF446902
	fwd2	CCAGGTAGCAAAAGGAAAGGC	
	rev1	GGAAGAAAGAAGCCTTAAACGAACC	
	rev2	CTTTGGAAGAACAGAGCATTATTTC	
*BCAR4 (v1+v2)*	fwd1	GAAGGGTGTTTGCTGATTTCTGTTAAG	EF446907
	fwd2	CCTGAAGGGTGTTTGCTGAT	
	rev1	CATTGTTGTTACCAGGGCGAAGG	
	rev2	CCAGGTCCACTGACTGTTAGC	
*BCAR4 (v1)*	fwd	TCTTGTCCATCGTCCTGTCCTG	EF446906
	rev	GTAGACCACACCATTACATCAGAGG	
*RNF18*	fwd1	TGCTGTGCTTGTTCTGCTCTC	EF446903
	fwd2	GCTTCTTTTGCTCTCTCTCATCTCCTC	
	rev1	GCTCGTATCATCACCAGTCGTAG	
	rev2	AATGGTGGCAGGGTCTGTGAA	
*STELLA (v1)*	fwd	TAGGACTACGCCCATTCACC	EF446904
	rev1	TGCTGTAGGCTCAAACTGCTC	
	rev2	GGGTCCAGGTTGGGTTATCT	
*STELLA (v2)*	fwd1	GCGGGGATGGCTACTCTTC	EF446905
	fwd2	TCTTCATCCCCTACAAAAGCA	
	rev1	TGCTGTAGGCTCAAACTGCTC	
	rev2	GGGTCCAGGTTGGGTTATCT	
*CCNB1*	fwd	TGGGTCGGCCTCTACCTTTGCACTTC	NM_001045872
	rev	CGATGTGGCATACTTGTTCTTGATAGTCA	
*CDC2*	fwd	ATGGCTTGGATCTGCTCTCG	NM_174016
	rev	CATTAAAGTACGGATGATTCAGTGC	
*PRDX1*	fwd	TCAAGCCTGATGTCCAGAAGAGC	NM_174431
	rev	CCGTCCTGTCCCACACCAC	
*PRDX2*	fwd	GATTATGGCGTGCTGAAGGAAGATG	NM_174763
	rev	GAGCGTCCCACAGGCAAGTC	
*ID2*	fwd	CAGTCCAGTGAGGTCCGTTAGG	AW464352
	rev	GCAGGCTCATCGGGTCGTC	
*ID3*	fwd	TGACGACATGAACCACTGCTACTC	BM106920
	rev	GCTGTCTGGATGGGAAGATGCG	
*AURKA*	fwd	TCGGGAGGACTTGGTTTCTT	DQ334808
	rev	TGTGCTTGTGAAGGAACACG	

### Reverse-transcription for real-time PCR

After adding 1 pg of luciferase mRNA (Promega, Charbonnières-les-bains, France) per oocyte/embryo as an exogenous standard, total DNAse-treated RNA was purified from 4 independent pools of 10 oocytes or embryos at each stage (or 5 to 16 OPU oocytes) using the Picopure RNA isolation kit (Alphelys, Plaisir, France). Reverse transcription was performed at 37°C for 50 min using oligo(dT)_15 _primers (Promega) or random hexamers (Promega) by mouse Moloney leukaemia virus reverse transcriptase (Invitrogen) onto RNA equivalent to 2.5 in vivo collected oocytes and 5 post-mortem collected oocytes or in vitro produced embryos.

### Real time PCR

Target cDNA were quantified by real-time PCR using iQ SYBR green supermix (Bio-Rad, Marnes la Coquette, France) with a iCycler or MyiQCycler system (Bio-Rad) using specific primers (sequences are presented in table 2). Primers for *CCNB1 *did not discriminate variants present in bovine oocytes[64]. A 3-step protocol (95°C for 30 sec, 60°C for 30 sec, 72°C for 20 sec) was repeated for 40 to 50 cycles, followed by acquisition of the melting curve. Amplicons were sequenced after cloning into pCRII vector using TA cloning kit (Invitrogen). The standard curve was deduced from serial dilutions of a plasmid including the target sequence, adjusted so that samples would fall within the considered range. Depending on the target gene, a cDNA amount equivalent to 0.0005 to 0.2 oocyte or embryo was used in triplicate PCR reactions, and the median value was considered (0 when not detected). For each sample, the data were normalized to the median value for exogenous luciferase. The gene/luciferase value in mature oocytes was then compared to this same ratio in immature oocytes (mean of four oocyte or embryo collections); data are presented as mean ± SEM. For each gene, Mann-Whitney comparison test was used to compare a) detection in mature vs immature oocytes, b) data obtained with random hexamers primed vs oligo(dT)_15 _primed RT products, c) regulation in in vitro matured vs in vivo matured oocytes. For embryonic development from mature oocytes onwards, after analysis of variance, differences were evaluated using Tukey comparison test. In all instances, differences were considered statistically significant at the 95% confidence level (P < 0.05).

## Authors' contributions

All authors were involved in biological material collection. AT participated in experimental design, data analysis, figure editing and carried out most experimental work: oocyte in vitro maturation, embryo production, RNA extraction, real-time PCR for oocyte marker genes, statistical analysis and characterization of oocyte markers MELK and RNF18. PP carried out real-time PCR experiments onto genes previously identified as oocyte maturation markers. CP carried out in vitro maturation and embryo production. SP characterized STELLA and BCAR4. SU and JMT supervised and participated in the study of AURKA. PH and CJ supervised and carried out animal ovarian stimulation and OPU. RDT designed and supervised the study, analysed data and wrote the manuscript. All authors have read and approved the final manuscript.
